# Cost-effectiveness of Brief Behavioral Therapy for Pediatric Anxiety and Depression in Primary Care

**DOI:** 10.1001/jamanetworkopen.2021.1778

**Published:** 2021-03-15

**Authors:** Frances L. Lynch, John F. Dickerson, Michelle S. Rozenman, Araceli Gonzalez, Karen T. G. Schwartz, Giovanna Porta, Maureen O’Keeffe-Rosetti, David Brent, V. Robin Weersing

**Affiliations:** 1 Center for Health Research, Kaiser Permanente Northwest, Portland, Oregon; 2 Department of Psychology, University of Denver, Denver, Colorado; 3 Department of Psychology, California State University of Long Beach, Long Beach; 4 Department of Psychology, University of Maryland, College Park, College Park; 5 Department of Psychiatry, University of Pittsburgh School of Medicine, Pittsburgh, Pennsylvania; 6 Joint Doctoral Program in Clinical Psychology, San Diego State University and University of California San Diego, San Diego; 7 Department of Psychology, San Diego State University, San Diego, California

## Abstract

**Importance:**

Youth anxiety and depression are common and undertreated. Pediatric transdiagnostic interventions for anxiety and/or depression may be associated with improved access to treatment among youths.

**Objective:**

To evaluate the cost-effectiveness of a pediatric transdiagnostic brief behavioral therapy (BBT) program for anxiety and/or depression compared with assisted referral to community outpatient mental health care (ARC).

**Design, Setting, and Participants:**

In this economic evaluation, an incremental cost-effectiveness analysis was performed from the societal perspective using data from a randomized clinical trial of youths with full or probable diagnoses of anxiety or depression who were recruited from pediatric clinics in San Diego, California, and Pittsburgh, Pennsylvania. The trial was conducted from October 6, 2010, through December 5, 2014, and this analysis was performed from January 1, 2019, through October 20, 2020.

**Interventions:**

In the randomized clinical trial, youths were randomized to BBT (n = 95) or ARC (n = 90). The BBT program consisted of 8 to 12 weekly 45-minute sessions of behavioral therapy delivered in pediatric clinics by master’s-level therapists. Families randomized to ARC received personalized referrals to mental health care and telephone calls to support access to care.

**Main Outcomes and Measures:**

Anxiety-free days, depression-free days, quality-adjusted life-years (QALYs), and costs based on incremental cost-effectiveness ratios from intake through 32-week follow-up. A cost-effectiveness acceptability curve for QALYs was used to assess the probability that BBT was cost-effective compared with ARC over a range of amounts that a decision-maker might be willing to pay for an additional outcome.

**Results:**

Enrolled patients included 185 youths (mean [SD] age, 11.3 [2.6] years; 107 [57.8%] female; 144 [77.8%] White; and 38 [20.7%] Hispanic). Youths who received BBT experienced significantly more anxiety-free days (difference, 28.63 days; 95% CI, 5.86-50.71 days; *P =* .01) and QALYs (difference, 0.026; 95% CI, 0.009-0.046; *P* = .007) compared with youths who received ARC. Youths who received BBT experienced more depression-free days than did youths who received ARC (difference, 10.52 days; 95% CI, −4.50 to 25.76 days; *P* = .18), but the difference was not statistically significant. The mean incremental cost-effectiveness ratio was −$41 414 per QALY (95% CI, −$220 601 to $11 468). The cost-effectiveness acceptability curve analysis indicated that, at a recommended willingness-to-pay threshold of $50 000 per QALY, the probability that BBT would be cost-effective compared with ARC at 32 weeks was 95.6%.

**Conclusions and Relevance:**

In this economic evaluation, BBT in primary care was significantly associated with better outcomes and a greater probability of cost-effectiveness at 32 weeks compared with ARC. The findings suggest that transdiagnostic BBT may be associated with improved youth anxiety and functioning at a reasonable cost.

## Key Points

**Question:**

Is transdiagnostic brief behavioral therapy in primary care cost-effective for treatment of youth anxiety and depression compared with assisted referral to community outpatient mental health care?

**Findings:**

In this economic evaluation of 185 youths with anxiety and/or depression who received brief behavioral therapy or assisted referral to community outpatient mental health care, brief behavioral therapy was associated with significantly more quality-adjusted life-years and a greater probability of cost-effectiveness at 32 weeks compared with assisted referral to outpatient care.

**Meaning:**

These results suggest that dissemination of effective transdiagnostic interventions for anxiety and depression may be associated with improved health among youths and with reduced costs.

## Teaser

‎This economic evaluation assessed the cost-effectiveness of a pediatric transdiagnostic brief behavioral therapy program for anxiety and/or depression in primary care compared with assisted referral to community outpatient mental health care.

## Introduction

Anxiety and depression during childhood and adolescence are common, highly comorbid, and associated with significant impairment.^1,2,3,4,5^ Approximately 30% of youths experience anxiety, depression, or both by the end of puberty.^1^ These disorders are associated with long-term negative outcomes, including impairment in relationships, poor academic performance, reduced quality of life, increased risk of anxiety and depression in adulthood, teen pregnancy, substance use, and self-harm.^3,4,5^

Several evidence-based treatments are available for youth anxiety and/or depression, including medication, cognitive behavioral therapy, and interpersonal psychotherapy.^6,7,8,9,10,11^ However, less than 50% of youths with these conditions receive any mental health service.^12^ Youths with anxiety and depression also often experience physical symptoms that require extensive testing and treatment and thus lead to avoidable costs. Without treatment, many youths continue to experience substantial burden, and increased severity and chronicity are associated with decreased treatment responsiveness and increased costs.^13,14^

Despite availability of efficacious treatments for anxiety and depression,^6,7,8,9,10,11^ usual care services often do not offer evidence-based approaches specifically targeting anxiety or depression.^15,16,17^ Health systems experience barriers to providing services with the strongest evidence base,^18^ including a lack of information about the immediate budgetary impact of introducing programs as well as their longer-term effects on costs.^18^ Furthermore, health systems must make decisions regarding which programs to select given that many evidence-based treatments are designed to manage only 1 disorder (eg, cognitive behavioral therapy for anxiety,^9^ interpersonal psychotherapy for depression^11^). Recent initiatives by the National Institute of Mental Health have stressed the importance of developing transdiagnostic interventions designed to target core processes common across frequently comorbid disorders and to improve their uptake in busy primary care clinics.^19^ Providing decision-makers with information on the costs and benefits associated with transdiagnostic programs may encourage expansion of effective services for youths. However, studies of the cost-effectiveness of any evidence-based mental health programs for youths are rare, and to our knowledge, there have been no economic evaluations of a transdiagnostic intervention for youths.

In this study, we performed a cost-effectiveness analysis (CEA) of a transdiagnostic brief behavioral therapy (BBT) program designed for youths with anxiety and/or depression diagnoses that was delivered in pediatric primary care settings.^17^ The BBT program was designed to target common core mechanisms of avoidance of negative effects and poor approaches to rewarding stimuli thought to be associated with anxiety and depression in youths. The BBT intervention components were based on youth anxiety (graded exposure) and depression (behavioral activation) research.^20^ Data for the CEA were from a randomized clinical trial (RCT)^20,21^ that evaluated the effects of BBT compared with assisted referral to community outpatient mental health care (ARC). In the RCT, BBT had significantly better clinical effects immediately after treatment and at follow-up assessments, and BBT was particularly effective for Hispanic youths. The present study assessed whether BBT is also a more cost-effective anxiety and depression treatment compared with ARC by using a CEA from a societal perspective from intake through the 32-week follow-up period.

## Methods

### Participants

This economic evaluation used data from an RCT^20,21^ of BBT vs ARC that enrolled 185 youths aged 8.0 to 16.9 years who met criteria for full or probable diagnoses of anxiety, depression, or both. Overall, 114 youths (61.6%) met the criteria for an anxiety disorder, of whom 60 (32.4%) had a diagnosis of clinically significant anxiety and depression and 11 (5.9%) had depression without current anxiety. Youths were excluded if they had a current *Diagnostic and Statistical Manual of Mental Disorders* (Fourth Edition) diagnosis of bipolar disorder or substance dependence or report of current suicidal plan, current abuse, intellectual disability or school performance below the second-grade level, or unstable serious physical illness. Also excluded were youths receiving active alternate treatment for anxiety or depression.^20,21^ The trial recruited participants from pediatric clinics in San Diego, California (4 sites), and Pittsburgh, Pennsylvania (5 sites), from October 6, 2010, to December 5, 2014. The current analysis was conducted from January 1, 2019, to October 20, 2020. The institutional review boards at San Diego State University, the University of Pittsburgh, and Kaiser Permanente of Southern California approved this study. We obtained written informed consent and assent from parents and youths, respectively. The study followed the Consolidated Health Economic Evaluation Reporting Standards (CHEERS) reporting guideline.

In the RCT, youths were randomly assigned to BBT (n = 95) or ARC (n = 90) with a balance of race/ethnicity and clinically significant depression at baseline.^21^ Brief behavioral therapy is a transdiagnostic intervention developed to address youth anxiety, depression, and comorbid presentation. It consisted of 8 to 12 weekly 45-minute sessions delivered in a primary care setting by a master’s-level therapist. The intervention was explicitly behavioral (vs cognitive-behavioral) and combined exposure and behavioral activation to improve engagement in avoided activities, alleviate symptoms, and improve functioning. The program also included brief training in relaxation to manage somatic symptoms and problem solving for stress management.

The comparison group (ARC) received telephone calls from project staff, assistance, and referrals to help with engagement in outpatient mental health services after randomization. Additional details about the trial design, intervention, and outcomes are reported elsewhere.^20,21^

### Clinical Outcomes

In the RCT, independent evaluators assessed youths’ current diagnoses using the Schedule for Affective Disorders and Schizophrenia for School-Age Children,^22^ and the Clinical Global Impressions Scale was used to assess global severity^23^ and improvement of anxiety and depression at baseline, 16 weeks, and 32 weeks after the intervention. Functional impairment was rated by independent evaluators using the Children’s Global Assessment Scale.^24^ Anxiety severity was assessed using the Pediatric Anxiety Rating Scale^25^ and depression severity with the Children’s Depression Rating Scale–Revised.^26^ We assessed health-related quality of life using the Health Utilities Index Mark 2 (HUI2) to calculate quality-adjusted life-years (QALYs) for the CEA.^27^

We created measures of QALYs,^28,29,30,31^ anxiety-free days (AFDs),^32,33,34^ and depression-free days (DFDs)^35,36^ based on CEA of other mental health treatments. To calculate AFDs and DFDs, we used the Pediatric Anxiety Rating Scale and Children’s Depression Rating Scale–Revised scores, respectively, from all follow-up points to categorize (1) days with no symptoms, (2) days with some symptoms but not meeting the full criteria for depression or anxiety disorders, and (3) days with symptoms meeting the diagnostic criteria for depression or anxiety disorders. To calculate symptoms for each day during the follow-up period, we used linear interpolation to assign a value to each day in the interval, with days with milder symptoms weighted less. The number of AFDs or DFDs was the total number of days in the interval minus the number of days with symptoms. This approach was used to capture the association of the intervention with the outcomes by including days with mild symptoms and days with significant symptoms.^35,36,37^

### BBT and ARC Intervention Costs

We used records from the RCT about activities, facilities, and overhead costs to calculate intervention costs. Paid master’s-level therapists, similar to staff who might provide the intervention in primary care, conducted the intervention in the RCT. They estimated time to complete intervention tasks as well as their use of equipment and supplies. We included costs of BBT sessions or ARC referral telecalls by project staff, time therapists and staff spent with youths and parents on the telephone or in person, clinical training, supervision, and materials. Research-specific costs (eg, randomization) were excluded.

### Usual Care Services

The CEA examined the incremental difference in clinical outcomes (QALYs, DFDs) and costs of the BBT intervention compared with ARC. We did not attempt to control the type of setting or payment model. We used the Child and Adolescent Services Assessment^38^ to gather data on usual care services for both the BBT and the ARC groups, capturing a wide range of health, social, and educational services. We applied nationally representative unit costs developed for previous studies.^35,36^ A youth and a parent separately reported services that the youth received. This method allowed us to gather information on the variety of services that youths are likely to use (eg, health system, school, and social welfare) without having to obtain data from multiple systems. Given that youths came from a wide range of community clinics and geographic areas, this method was the most practical for obtaining comparable data for all youths. For the baseline assessment, youths reported services received in the previous 3 months; at each follow-up assessment, they reported services received since the previous assessment.

### Family Costs

In accordance with expert guidelines for economic evaluations,^39^ we estimated family costs as the time that parents spent bringing youths to services. We created profiles of usual care service use and then estimated parent time spent in services, travel to services, and waiting time based on prior research.^35,36,40^

### Statistical Analyses

We took a societal perspective when estimating incremental cost-effectiveness ratios (ICERs). The time frame was from intake through 32-week follow-up; we were constrained to this time frame by the clinical trial design.^20^ We computed the ICERs as the mean cost difference between BBT and ARC divided by the mean difference in clinical outcome (eg, QALYs). All costs were adjusted for inflation. We used the net benefit regression method^41^ with ordinary least-squares regression. We confirmed the robustness of parametric tests using nonparametric bootstrapping^42^ with a single model with 1000 replications using the bias-corrected and accelerated method.^43^ We estimated adjusted differences between groups using ordinary least-squares regression models with bootstrap interval estimates. All costs are shown in 2014 US dollars. We adjusted for site and baseline values when appropriate.

**Figure 1.**
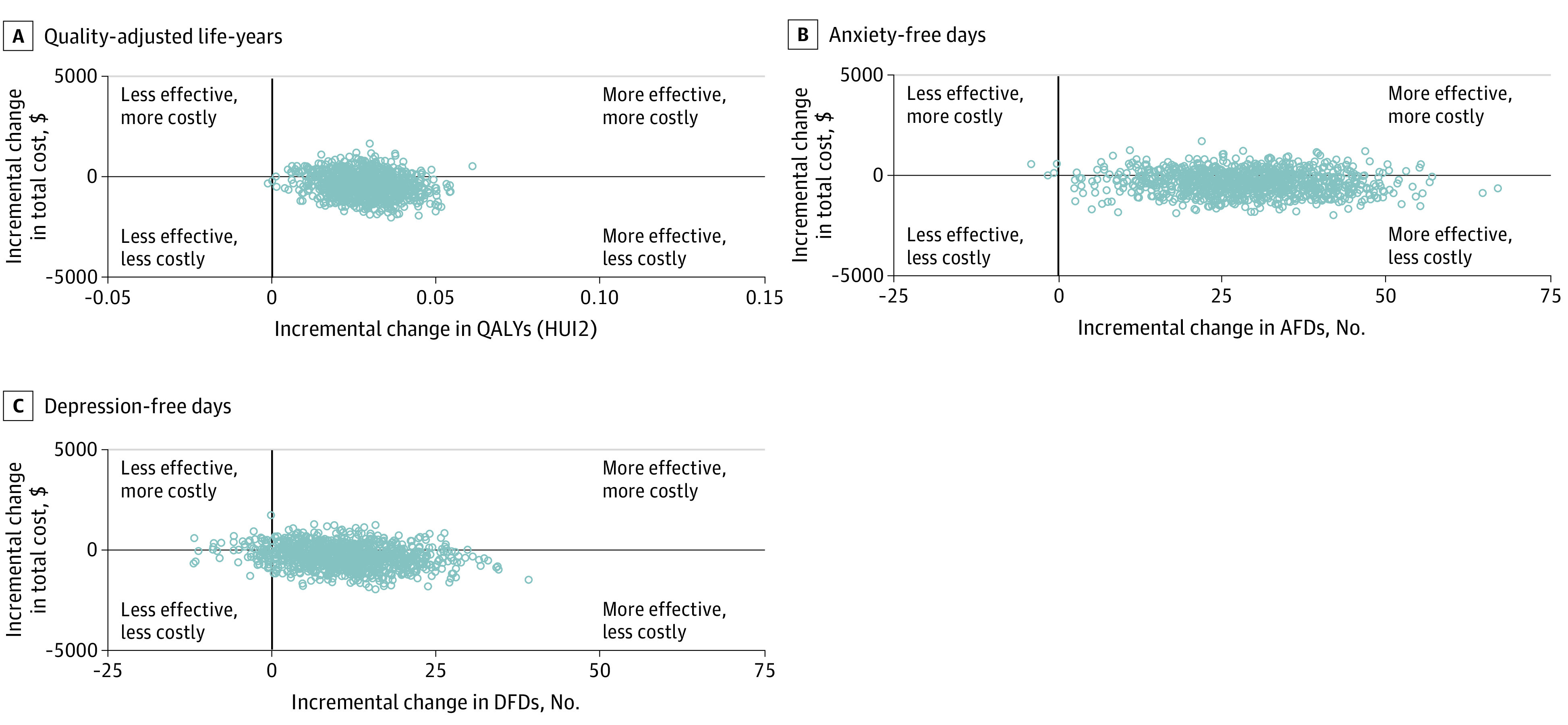
Cost-effectiveness Planes Scatter plots of bootstrapped cost-and-effect pairs were used to construct cost-effectiveness planes divided into 4 quadrants centered at 0. If the majority of the cost-effect observations were located in the SE quadrant the program was considered to be less costly and more effective. AFDs indicate anxiety-free days; DFDs, depression-free days; HUI2, Health Utilities Index Mark 2; QALYs, quality-adjusted life-years.

**Figure 2.**
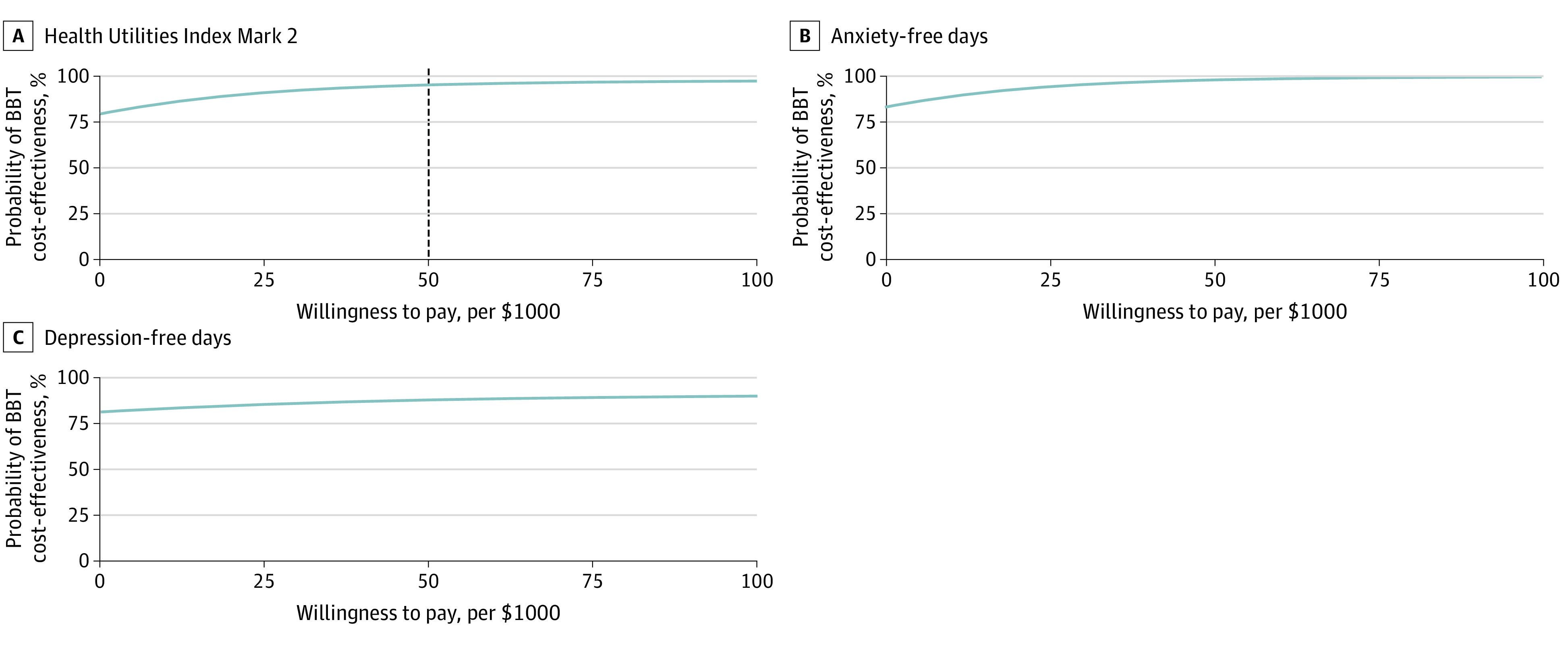
Cost-effectiveness Acceptability Curves The probability that brief behavioral therapy (BBT) was cost-effective compared with assisted referral to community outpatient mental health care over a range of amounts that a decision-maker might be willing to pay for an additional outcome. Vertical dashed line indicates the reasonable threshold for cost-effectiveness of $50 000 or less per quality-adjusted life-year.

We used bootstrap observations to estimate a 95% CI around the mean ICER to represent uncertainty in the estimates. We next created scatter plots of bootstrapped cost-and-effect pairs to construct cost-effectiveness planes^44^ divided into 4 quadrants centered at 0 (Figure 1). If the majority of the cost-and-effect observations were located in the southeast quadrant, the program was considered to be less costly and more effective. A cost-effectiveness acceptability curve for QALYs was used to assess the probability that BBT was cost-effective compared with ARC over a range of amounts that a decision-maker might be willing to pay for an additional outcome (Figure 2). Statistical significance was set at a 2-sided α = .05.

#### Supplemental Analyses

We also conducted supplemental analyses to examine the robustness of results. Clinical outcomes from the RCT suggested that although BBT was effective overall, it was particularly effective for certain groups and outcomes.^20,21^ Hispanic youths had greater response to BBT across metrics, and depression trajectories were similar for BBT and ARC, with both treatment groups demonstrating improvement over time but neither treatment demonstrating superiority. We thus examined whether these factors moderated cost-effectiveness. We conducted subgroup analyses based on the moderating variables found in the main RCT. In addition, we tested the interaction between the potential moderating variable and treatment condition in the intention-to-treat sample.

#### Missing Data

Overall, 84.9% of youths completed the 32-week Child and Adolescent Services Assessment. We imputed missing data using multiple imputation with chained equations in Stata, version 15.1 (StataCorp LLC).^45,46^ We included baseline demographic characteristics, functioning (Children’s Global Assessment Scale score), and depression level (Children’s Depression Rating Scale–Revised score) as well as all nonmissing values of costs or outcomes at all time points in the imputation models.^21,46^ We created 10 imputation data sets and combined estimates so that SEs reflected the variability introduced by the imputation process.^46^

## Results

Enrolled patients included 185 youths (mean [SD] age, 11.3 [2.6] years; 107 [57.8%] female; 144 [77.8%] White; and 38 [20.7%] Hispanic). As reported elsewhere,^20,21^ baseline demographic and clinical characteristics did not differ significantly by group.^20,21^ Baseline usual care costs also did not differ significantly between groups.

### Clinical Outcomes

In the base-case CEA, compared with youth who received ARC, those who received BBT experienced more AFDs (difference, 28.63 days; 95% CI, 5.86-50.71 days; *P* = .01) and QALYs (difference, 0.026; 95% CI, 0.009-0.046; *P* = .007) at 32 weeks. Youths who received BBT experienced more DFDs than did youths who received ARC (difference, 10.52 days; 95% CI, −4.50 to 25.76 days; *P* = .18), but the difference was not statistically significant.

### Service Use and Costs

Table 1 and Table 2 show usual care service use by study condition and the mean (SD) costs of usual care and intervention services by condition, respectively. The mean (SD) cost of delivering the 12-session BBT intervention was significantly greater than the mean (SD) cost of ARC ($1183 [$341] vs $32 [$54]; *P* < .001). By design, youths in ARC were encouraged to use usual care services. The mean (SD) cost of usual care services was $2152 lower for BBT than for ARC ($2735 [$4708] vs $4887 [$9088]; *P* = .01). The difference in the mean total cost between BBT and ARC was –$1082 (95% CI, –3325 to 289; *P* = .24) over the 32-week period; this difference was not statistically significant.

**Table 1.** Usual Care Service Use From Intake Through 32 Weeks’ Follow-up^a^

Usual care service	BBT group (n = 95)	ARC group (n = 90)
Inpatient mental health care		
Patients, No. (%)	1 (1.2)	1 (1.3)
Duration, mean (SD), d	7.0 (0)	24.0 (0)
Inpatient alcohol or drug use	0	0
Counseling or medication management		
Patients, No. (%)	21 (25.9)	65 (85.5)
Visits, mean (SD)	1.7 (1.0)	2.8 (1.2)
Hospital stay	0	0
Alcohol or drug treatment visits	0	0
Crisis services		
Patients, No. (%)	0	2 (2.6)
Services, mean (SD)	NA	1.5 (0.7)
Medical doctor visit		
Patients, No. (%)	34 (42.0)	26 (34.2)
Visits, mean (SD)	1.5 (0.7)	1.3 (0.6)
Emergency department visit		
Patients, No. (%)	14 (17.3)	18 (23.7)
Visits, mean (SD)	1.3 (0.5)	1.2 (0.4)
Antidepressant medication use		
Patients, No. (%)	5 (6.2)	8 (10.5)
Duration, mean (SD), d	252.0 (184.4)	157.5 (63.6)
Stimulant medication use		
Patients, No. (%)	6 (7.4)	5 (6.6)
Duration, mean (SD), d	165.0 (88.5)	90.0 (0.0)
Psychotropic medication use	0	0
Any school services		
Patients, No. (%)	58 (71.6)	53 (69.7)
Services, mean (SD)	32.3 (59.2)	38.0 (67.1)
Juvenile correction contact		
Patients, No. (%)	0	1 (1.3)
Contacts, mean (SD)	NA	1.0 (0)

Abbreviations: ARC, assisted referral to community outpatient mental health care; BBT, brief behavioral treatment; NA, not applicable.

^a^ Analyses of use of services were unadjusted.

**Table 2.** Usual Care Costs From Intake Through 32 Weeks’ Follow-up

Type of care	Cost, mean (SD), $^a^	
	BBT group	ARC group
Usual care services cost	2735 (4708)	4887 (9088)
Intervention		
Program costs	1183 (341)	32 (54)
Intervention family costs	39 (24)	3 (4)
Total cost per participant	3957 (4074)	4923 (9117)

Abbreviations: ARC, assisted referral to community outpatient mental health care; BBT, brief behavioral treatment.

^a^ Costs were imputed when data were missing.

### Cost-effectiveness Analysis

Table 3 describes the clinical outcomes, the mean cost by group, and the ICERs. At 32 weeks, the estimated cost per AFD was –$38 (ICER, –$38; 95% CI, –$224 to $1), the estimated cost per DFD was −$103 (ICER, –$103; 95% CI, –$4705 to $107), and the estimated cost per QALY was –$41 414 (ICER, –$41 414; 95% CI, –$220 601 to $11 468). In all cases, the base-case analysis indicated that BBT was cost saving on average.

**Table 3.** Incremental Adjusted Differences and ICERs From Intake Through 32 Weeks’ Follow-up

Dependent variable	BBT group	ARC group	Difference (95% CI)^a^^,^^b^	*P* value	ICER, $ (95% CI)^b^^,^^c^
Cost outcome					
Total costs, $	3957 (4074)	4923 (9117)	–1082 (–3324.80 to 289.44)	.24	NA
Clinical outcome					
QALYs^d^	0.455 (0.078)	0.427 (0.106)	0.026 (0.009 to 0.046)	.007	–41 414 (–220 601 to 11 468)
Anxiety-free days	135.8 (79.3)	107.1 (87.9)	28.63 (5.86 to 50.71)	.01	–38 (–224 to 13)
Depression-free days	195.4 (54.4)	184.9 (61.8)	10.52 (–4.50 to 25.76)	.18	–102 (–4705 to 107)

Abbreviations: ARC, assisted referral to community outpatient mental health care; BBT, brief behavioral treatment; NA, not applicable; QALYs, quality-adjusted life-years.

^a^ Adjusted for site and baseline values for costs.

^b^ Bias-corrected 95% CIs.

^c^ Computed as the mean cost difference between BBT and ARC divided by the mean difference in clinical outcome (QALYs, anxiety-free days, and depression-free days). All costs were adjusted for inflation.

^d^ QALYs calculated from the Health Utilities Index Mark 2.

Figure 1 shows the cost-effectiveness planes for the base-case analysis. Each point in the scatter plots represents a cost–clinical outcome pair for a single bootstrap replication. For all 3 outcomes (AFDs, DFDs, and QALYs) most of the observations (880 of 1000 observations [88%] for AFDs, 820 of 1000 [82%] for DFDs, and 880 of 1000 [88%] for QALYs) indicated better outcomes and lower costs at 32 weeks.

Figure 2 shows the cost-effectiveness acceptability curve for QALYs at 32 weeks for the base-case analysis. For example, if a decision-maker paid $50 000 per QALY, the probability that BBT would be cost-effective compared with ARC at 32 weeks was 95.6% (956 of 1000 replications).

### Supplemental Analyses

Based on the original RCT results, we tested whether Hispanic ethnicity or youth depression status at baseline moderated the cost-effectiveness of BBT. Results of the moderation analyses were not statistically significant: ethnicity (for cost: coefficient, $943 [95% CI, −$5156 to $3270]; *P* = .67; for QALYs: coefficient, −0.034 [95% CI, −0.077 to 0.009]; *P* = .55) and depression (for cost: coefficient, $676 [95% CI, −$3825 to $2473]; *P* = .66; for QALYs: coefficient, 0.013 [95% CI, −0.034 to 0.060]; *P* = .12). These findings suggest that results were broadly robust among these factors. We conducted further exploratory analyses to examine whether cost-effectiveness varied by health system payer and family perspectives and excluding factors strongly associated with cost-effectiveness (eTable in the Supplement). Among the models, results indicated some variation in clinical significance and cost per QALY for different assumptions. However, in all cases, the upper limit of the 95% CI around the ICER indicated that the cost per QALY was below the threshold of $50 000 per QALY.

## Discussion

This economic evaluation found that the cost of implementing BBT was low (mean [SD], $1183 [$341] per youth) but significantly greater than the cost of implementing ARC ($32 [$54] per youth). Furthermore, in the base-case CEA analyses, BBT was cost-saving on average, suggesting that BBT may be associated with improved outcomes and is cost-effective by most published standards.^47^ Our results build on prior research suggesting that youths with anxiety and/or depression can be successfully treated with a brief transdiagnostic psychosocial intervention.^6,7,8,9,10,11^ Although these results are promising, to our knowledge, few evidence-based interventions for either anxiety or depression are available in health care settings.^15,16,17^ One barrier has been a lack of information about the budgetary impact of programs and their long-term effects on health care costs.^18^

Several other evaluations have found that psychotherapeutic interventions for youth anxiety and depression are cost-effective.^28,29,30,31,48,49,50,51,52^ All of these studies examined interventions that addressed either anxiety^28,29,30,31^ or depression^50,51^ but not both, and most were conducted outside the US.^28,29,30,31^ The most comparable studies examined the cost-effectiveness of individual psychotherapeutic interventions for anxiety^30^ or depression^50^ and reported similar costs of BBT ($1000-$1500). To our knowledge, no previous studies have examined the cost-effectiveness of a transdiagnostic intervention aimed at treating anxiety, depression, or both.

Transdiagnostic interventions are attractive in busy primary care settings, where training, staffing, and offering separate interventions may be challenging.^15,16,17,18^ An intervention that addresses both conditions may help increase the availability of treatment from the health system and family perspectives. Depression status at baseline did not significantly moderate our base-case results, supporting the cost-effectiveness of BBT for the outcomes of QALYs and AFDs. Cost-effectiveness for depression-specific outcomes is less clear because depression symptoms in both the BBT and the ARC groups improved and trajectories were not significantly different. Our results provide support for use of transdiagnostic interventions in primary care because BBT had improved or similar associations with a range of outcomes compared with usual care services.

This study was designed to assess treatment options for pediatric primary care for several reasons. Primary care is the most common setting in which children are seen,^53,54^ and pediatric health care professionals are often trusted by parents and youths; this trust maybe be associated with reduced stigma related to initiating care among youths with anxiety and/or depression.^55,56^ Prior research suggests that delivering mental health care in primary care may be particularly important for publicly insured children or for those from non-White racial/ethnic groups.^20,21^ The RCT^20,21^ on BBT vs ARC indicated a significantly greater clinical effect of BBT on Hispanic youths compared with all other youths. We did not find significant moderation by ethnicity in the CEA likely owing to low power; however, BBT was cost-effective on average among racial/ethnic groups.

The primary analysis was conducted from the societal perspective.^39^ However, other perspectives may be important. Results of the supplemental analyses from the health system payer perspective also indicated cost savings associated with the BBT intervention. In addition, the ARC comparison condition was designed to encourage families to engage in usual care and included multiple telephone calls to overcome barriers; comparison families engaged in usual care at high levels.^12^ For this reason, we believe that access to care was likely not the reason for the differences in outcomes between the BBT and ARC groups. One possibility is that BBT is focused on skills that more directly address symptoms compared with typical treatment in usual care settings.^9,11^

### Limitations

This study has limitations. Although the study took place in 2 geographic areas (San Diego, California, and Pittsburgh, Pennsylvania), additional research is needed to better understand the generalizability of our results. Anecdotally, the BBT intervention was well received by health care professionals; however, health care professionals’ satisfaction was not assessed, nor were reasons that clinics declined participation. We collected data on usual care services using self-report, which is subject to recall bias. We tried to minimize this concern by using a psychometrically sound instrument (the Child and Adolescent Services Assessment)^38^ used in prior studies^35,36,40^ and by using trained interviewers. We did not systematically test whether BBT was more cost-effective than delivering separate anxiety and depression interventions. The study period was brief; studying longer-term outcomes is an important next step. We used nationally representative unit costs to estimate costs of usual care service; these may not reflect costs at study sites.

## Conclusions

In this economic evaluation, BBT in primary care was significantly associated with better outcomes and a greater probability of cost-effectiveness at 32 weeks compared with ARC. Results of this study suggest that dissemination of effective transdiagnostic interventions for anxiety and depression may be associated with improved health among youths and with reduced costs to the health care system and society.

## Notes

Supplement. **eTable.** Sensitivity Analyses Around ICER Comparing BBT to ARC From Intake Through 32 Weeks Follow-Up
