# Supplementary material for: Cost-effectiveness of Brief Behavioral Therapy for Pediatric Anxiety and Depression in Primary Care
Source: JAMA Netw Open. 2021 Mar 15;4(3):e211778. doi: 10.1001/jamanetworkopen.2021.1778 (PMC7961309; doi:10.1001/jamanetworkopen.2021.1778)
Supplement: Supplement. — eTable. Sensitivity Analyses Around ICER Comparing BBT to ARC From Intake Through 32 Weeks Follow-Up [file jamanetwopen-e211778-s001.pdf]

Supplemental Online Content

Lynch FL, Dickerson JF, Rozenman MS, et al. Cost-effectiveness of brief behavioral therapy for pediatric anxiety and depression in primary care. *JAMA Netw Open.* 2021;4(3):e211778. doi:10.1001/jamanetworkopen.2021.1778

eTable. Sensitivity Analyses Around ICER Comparing BBT to ARC From Intake Through 32 Weeks Follow-Up

This supplemental material has been provided by the authors to give readers additional information about their work.

© 2021 Lynch FL et al. *JAMA Network Open.*

eTable. Sensitivity Analyses around ICER Comparing BBT to ARC from Intake thru 32 weeks Follow-Up

**Sensitivity Analysis**	Difference[1] (BBT vs ARC)	*p-value*	ICER	95% CI[2]	
				Lower	Upper
**Health System Payer Perspective**					
Cost					
Total Costs (USD)	**-$500**	*0.526*			
Clinical Outcome					
QALYs[3]	**0.026**	*0.007*	**-$19,019/QALY**	-153,623	30,407
Anxiety-Free Days	29	*0.010*	**-$17/AFD**	-114	30
Depression-Free Days	10	*0.213*	**-$51/DFD**	-1,119	216
**Family Perspective**					
Cost					
Total Costs (USD)	**$7**	*0.531*			
Clinical Outcome					
QALYs[3]	**0.026**	*0.007*	**$252/QALY**	-1,150	1,268
Anxiety-Free Days	29	*0.010*	**0$/AFD**	-1	1
Depression-Free Days	11	*0.184*	**$1/DFD**	-6	19
**Overly Influential Outliers Removed**					
Cost					
Total Costs (USD)	**-$435**	*0.448*			
Clinical Outcome					
QALYs[3]	**0.028**	*0.003*	**-$15,349/QALY**	*-74,930*	*28,820*
Anxiety-Free Days	**29**	*0.007*	**-$15/AFD**	*-87*	*27*
Depression-Free Days	**11**	*0.156*	**-$40/DFD**	*-970*	*120*
**Minority Youth**					
Cost					
Total Costs (USD)	**-$1,677**	*0.170*			
Clinical Outcome					
QALYs[3]	**0.048**	*0.023*	**-$34,841/QALY**	*-166,374*	*11,974*
Anxiety-Free Days	**42**	*0.075*	**-$40/AFD**	*-403*	*59*
Depression-Free Days	**22**	*0.072*	**-$77/DFD**	*-606*	*74*
**Non-Minority Youth**					
Cost					
Total Costs (USD)	**-$1,002**	*0.376*			
Clinical Outcome					
QALYs[3]	**0.020**	*0.065*	**-$50,098/QALY**	*-1,114,345*	*39,301*
Anxiety-Free Days	**26**	*0.032*	**-$38/AFD**	*-360*	*31*
Depression-Free Days	**8**	*0.368*	**-$120/DFD**	*-6,997*	*132*
**No Depression at Baseline (Anxiety Only)**					

© 2021 Lynch FL et al. *JAMA Network Open.*

Cost					
Total Costs (USD)	**-$734**	*0.342*			
Clinical Outcome					
QALYs[3]	**0.035**	*0.003*	**-$20,808/QALY**	*-88,746*	*26,387*
Anxiety-Free Days	**38**	*0.005*	**-$19/AFD**	*-88*	*27*
Depression-Free Days	**8**	*0.078*	**-$96/DFD**	*-743*	*312*
**Depression and Anxiety at Baseline**					
Cost					
Total Costs (USD)	**-$1,670**	*0.480*			
Clinical Outcome					
QALYs[3]	**0.024**	*0.511*	**-$70,829/QALY**	*-8,450,007*	*31,453*
Anxiety-Free Days	**8**	*0.619*	**-$199/AFD**	*-1,250,240*	*23*
Depression-Free Days	**19**	*0.234*	**-$86/DFD**	*-3,811*	*221*
**Depression at Baseline (All Subjects with Depression at Baseline)**					
Cost					
Total Costs (USD)	**-$1,677**	*0.405*			
Clinical Outcome					
QALYs[3]	**0.011**	*0.515*	**-$145,983/QALY**	*-1,850,007*	*39,742*
Anxiety-Free Days	**13**	*0.457*	**-$126/AFD**	*-7,541*	*169*
Depression-Free Days	**13**	*0.363*	**-$131/DFD**	*-9,770*	*47*

[1]BBT minus ARC; adjusted for site and baseline values. [2]Bias-corrected confidence interval. [3]QALYs from the HUI Mark 2

© 2021 Lynch FL et al. *JAMA Network Open.*
